# Fully automated preoperative segmentation of temporal bone structures from clinical CT scans

**DOI:** 10.1038/s41598-020-80619-0

**Published:** 2021-01-08

**Authors:** C. A. Neves, E. D. Tran, I. M. Kessler, N. H. Blevins

**Affiliations:** 1 Faculty of Medicine, University of Brasilia, Brasília, DF Brazil; 2 Otolaryngology Head & Neck Surgery, Stanford University School of Medicine, Stanford, CA USA

**Keywords:** Computed tomography, Health care, Surgery

## Abstract

Middle- and inner-ear surgery is a vital treatment option in hearing loss, infections, and tumors of the lateral skull base. Segmentation of otologic structures from computed tomography (CT) has many potential applications for improving surgical planning but can be an arduous and time-consuming task. We propose an end-to-end solution for the automated segmentation of temporal bone CT using convolutional neural networks (CNN). Using 150 manually segmented CT scans, a comparison of 3 CNN models (AH-Net, U-Net, ResNet) was conducted to compare Dice coefficient, Hausdorff distance, and speed of segmentation of the inner ear, ossicles, facial nerve and sigmoid sinus. Using AH-Net, the Dice coefficient was 0.91 for the inner ear; 0.85 for the ossicles; 0.75 for the facial nerve; and 0.86 for the sigmoid sinus. The average Hausdorff distance was 0.25, 0.21, 0.24 and 0.45 mm, respectively. Blinded experts assessed the accuracy of both techniques, and there was no statistical difference between the ratings for the two methods (p = 0.93). Objective and subjective assessment confirm good correlation between automated segmentation of otologic structures and manual segmentation performed by a specialist. This end-to-end automated segmentation pipeline can help to advance the systematic application of augmented reality, simulation, and automation in otologic procedures.

## Introduction

Safe and effective middle- and inner-ear surgery requires extensive training and knowledge of radiological and surgical anatomy. Procedures such as cochlear implantation, tympanomastoidectomy, and superior semicircular canal dehiscence repair depend on the pre- and intra-operative identification of critical structures and an appreciation of their complex interrelationships^1^. Individualized preoperative planning and the implementation of augmented reality systems may assist in such surgery given the intricacy and variability of anatomy involved. Such efforts require specialized anatomical and radiological knowledge of the key structures, which takes considerable time and effort to acquire. A method for the rapid and accurate generation of patient-specific, high-fidelity 3D models for preoperative planning^2^ and intraoperative navigation^3,4^ would offer a variety of potential benefits to both patient and surgeon.

**Figure 1:**
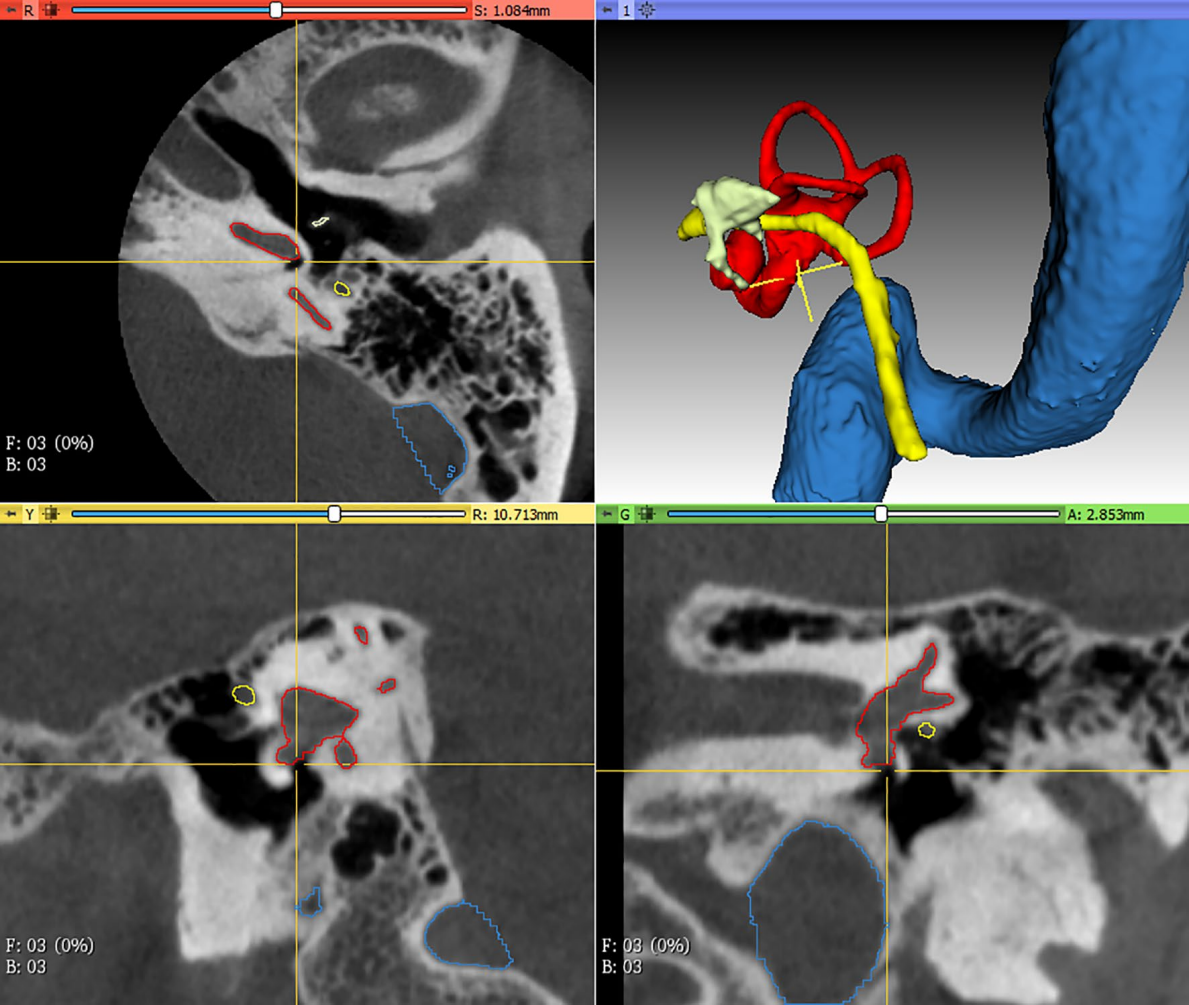
Manual segmentation of temporal bone structures as seen in 3D Slicer: inner ear (red), ossicles (ivory); facial nerve (yellow), sigmoid sinus (blue). Crosshair is centered over the round window niche.

Computed tomography (CT) imaging of the temporal bone is critical to provide otologists insights into a patient's unique anatomy for pre-operative planning. However, identifying structures of interest and subtle developmental or pathologic variations may be challenging for both surgeons and radiologists due to the structures’ small size and inherent complexity. However, understanding their orientation and geometry is essential for successful otologic procedures such as cochlear implantation or tumor removal^5^. In addition, although CT datasets are inherently volumetric, surgeons routinely review them as multiplanar two dimensional (2D) representations. This necessitates a mental translation of the data back into the three-dimensional (3D) relationships expected at the time of surgery. Efforts to enhance preoperative planning using innovative tools such as 3D simulations and augmented reality offer promise for improving operative safety and efficiency. However, these efforts are limited by the labor intensive step of manual segmentation of imaging data^6,7^ by highly trained specialists (Fig. 1). An automated pipeline of medical image segmentation for temporal bone CT (TBCT) scans might expand the application of simulation, planning, and procedural automation.

Cochlear implantation is an example of an otologic procedure that is both commonly performed and highly influenced by anatomic variability. As such, it has motivated a number of studies to integrate computer-assisted segmentation to increase safety and efficacy. Early works include computer-aided analysis of human temporal bone histopathology specimens by Nakashima et al.^8^. Noble et al.^9–11^ published a series of papers using atlas-based approaches and other customized solutions for automated identification of the facial nerve, ossicles and intracochlear anatomy. Recently, Powell et al.^12^ and Gare^5^ also showed strong correlation of atlas-based auto-segmentation of the temporal bone with ground truth. Hudson et al. used atlas-based models registered to micro-CT scans and manually placed fiducials to identify facial nerves in cadavers^13^. However, these methods require significant manual input, and therefore may be limited in their scalability and subject to user-dependent variability.

Deep learning (DL) techniques have been successfully implemented in a number of methods for automated identification of structures and lesions on CT or magnetic resonance (MR) images^14^. Fauser et al.^15^ described an automated mixed 2-dimensional deep learning with shape regularization approach to predict successful trajectories to the round window and to the internal auditory canal as a validation method. Algorithms based on convolutional neural networks have continually been improved for better prediction and faster implementation^16–18^. Efforts to connect the medical research community to the state-of-the-art machine learning tools such as NVIDIA Clara SDK^19^ and Slicer Artificial Intelligence Assisted Annotation (AIAA) Extension^20^ should leverage the development of new diagnostic and treatment techniques.

We describe a completely automated pipeline of computer-generated segmentation of key structures of the temporal bone derived from CT datasets. This uses a DL model trained on a dataset manually segmented by an expert. This approach offers potential benefits to enhanced radiological diagnosis and preoperative planning for a wide variety of otologic procedures.

## Results

The inner ear, facial nerve, ossicles, and sigmoid sinus were segmented manually by an expert in 150 de-identified TBCT (Fig. 1). The clinical evaluation of the dataset showed normal scans in 74%, post-operative changes in 9.3%, inflammatory or dysventilation related findings in 11.3%, and superior semicircular canal dehiscence in 2.7% (Table 1).

**Table 1:** Radiological findings from 150 temporal bone computed tomography scans (*Indicates overlapping of different procedures as well as inflammatory or dysventilation related changes in some sets).

	Absolute	Relative (%)
Normal	111	74.0
Stapedotomy	4	2.7
Ossiculoplasty	2	1.3
Middle ear prosthesis (PORP/TORP)	2	1.3
Mastoidectomy	5	4.0
Cochlear implant	1	0.7
Total post-operative	14*	9.3
Opacification of middle ear	9	6.0
Opacification of mastoid cells	5	3.3
Sclerotic mastoid	7	4.7
Total inflammatory or dysventilation findings	17*	11.3
SSC dehiscence	4	2.7
Others	4	2.0

*PORP* Partial ossicular reconstruction prosthesis, *TORP* Total ossicular reconstruction prosthesis, *SSC* Superior semicircular canal.

**Figure 2:**
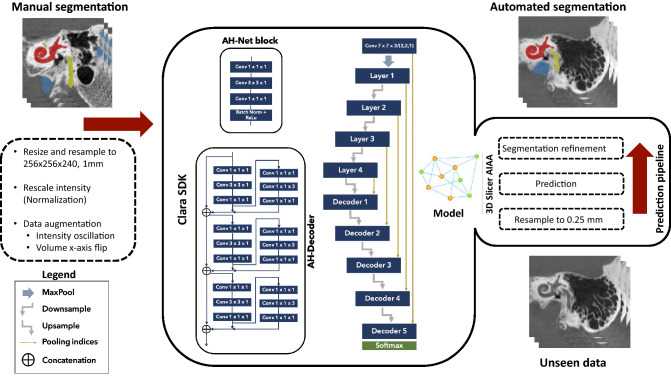
Auto-segmentation pipeline. Manual segmentation of temporal bone structures from computed tomography were used to train a deep learning model using a 3D convolutional neural network. Automated segmentation of the structures was then performed on a testing set in the 3D Slicer platform.

**Figure 3:**
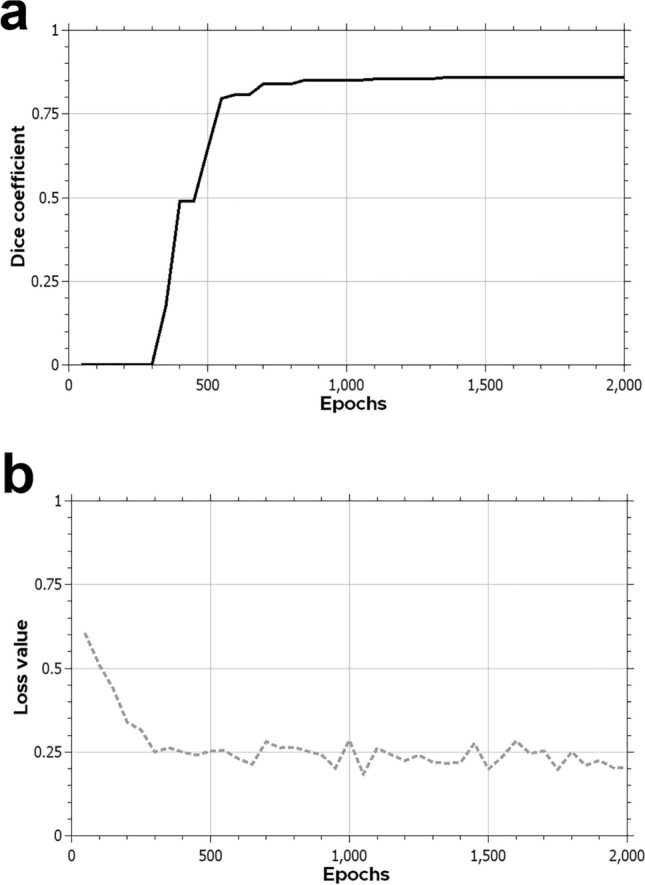
Training Dice similarity coefficient (**a**) and loss (**b**) for the inner ear training. The DSC graph demonstrates the improvement and eventual optimization of the model and the minimization of the loss function.

The reference TBCT volumes linked to the respective manually derived ground truth labels of each structure were successfully used to train the DL algorithm (Fig. 2) using AH-Net as well as 3D U-Net^21^ and 3D ResNet architectures^22^. At the completion of training, the with-in model prediction accuracy, measured by the Dice similarity coefficient (DSC) over the training dataset was 0.86 ± 0.08 (DSC ± standard deviation, SD) for the inner ear (Fig. 3); 0.77 ± 0.11 for the facial nerve; 0.84 ± 0.07 for the ossicles; and 0.86 ± 0.09 for the sigmoid sinus (Table 2).

**Table 2:** Mean Dice similarity coefficients from the training (cross-validation) and testing using different CNN architectures. SD: Standard deviation.

	Inner ear	Ossicles	Facial nerve	Sigmoid sinus
**Training (n = 125)**				
ResNet (SD)	0.90 (0.05)	0.84 (0.06)	0.70 (0.07)	0.79 (0.10)
U-Net (SD)	0.89 (0.05)	0.86 (0.07)	0.73 (0.07)	0.73 (0.11)
AH-Net (SD)	0.86 (0.08)	0.84 (0.07)	0.77 (0.11)	0.86 (0.09)
**Testing (n = 25)**				
ResNet (SD)	0.91 (0.03)	0.84 (0.10)	0.70 (0.07)	0.78 (0.09)
U-Net (SD)	0.89 (0.04)	0.84 (0.11)	0.71 (0.10)	0.74 (0.06)
AH-Net (SD)	0.88 (0.04)	0.86 (0.06)	0.71 (0.18)	0.83 (0.09)

To validate the models developed from the training data, we performed automated segmentation over the testing set (n = 25) using the NVIDIA Clara extension, which has been integrated with 3D Slicer. Using the automated prediction pipeline on this dataset produced a DSC of 0.91 ± 0.03 (DSC ± standard deviation, SD) for the inner ear; 0.75 ± 0.06 for the facial nerve; 0.85 ± 0.12 for the ossicles; and 0.86 ± 0.05 for the sigmoid sinus. The average Hausdorff distance (AHD) was 0.25, 0.21, 0.24 and 0.45 mm respectively, and the volumetric comparison had an average of 101% ± 23%. The results are displayed in Table 3.

**Table 3:** Results of objective validation of the automated segmentation of the testing dataset using the prediction pipeline (n = 25).

	Inner ear	Ossicles	Facial nerve	Sigmoid sinus
**Dice coefficient**				
ResNet (SD)	0.91 (0.03)	0.87 (0.04)	0.69 (0.11)	0.85 (0.04)
U-Net (SD)	0.91 (0.04)	0.86 (0.06)	0.73 (0.07)	0.81 (0.05)
AH-Net (SD)	0.91 (0.03)	0.85 (0.12)	0.75 (0.06)	0.86 (0.05)
**Time for segmentation (s)**				
Manual (SD)	224.2 (54.6)	110.3 (19.2)	221.8 (59.1)	323.3 (100.0)
ResNet (SD)	4.58 (0.52)	4.75 (0.62)	4.83 (0.56)	4.64 (0.55)
U-Net (SD)	6.82 (0.74)	6.72 (0.69)	6.84 (0.77)	6.71 (0.71)
AH-Net (SD)	2.61 (0.82)	2.70 (0.61)	2.73 (0.66)	2.65 (0.73)
**Hausdorff’s distance (mm)**				
ResNet (SD)	0.23 (0.18)	0.23 (0.18)	0.46 (0.42)	0.45 (0.15)
U-Net (SD)	0.25 (0.21)	0.22 (0.16)	0.38 (0.20)	0.62 (0.21)
AH-Net (SD)	0.25 (0.24)	0.23 (0.14)	0.24 (0.19)	0.45 (0.16)
**Volumetric similarity (%)**				
ResNet (SD)	104.8 (23.0)	101.2 (14.2)	108.7 (34.8)	104.7 (14.7)
U-Net (SD)	108.5 (13.2)	90.1 (11.9)	105.0 (32.9)	100.3 (25.1)
AH-Net (SD)	108.3 (13.3)	99.4 (30.9)	101.2 (24.3)	96.3 (18.9)

*SD* Standard deviation.

The time required for expert segmentation of the relevant anatomical structures by a trained otologist, experienced in the segmentation task was on average 211.1 s (Table 3). With the automated method, the key structures were segmented at an average of 2.7 s per structure. The automated otic capsule segmentation took an average of 2.2 s of processing.

An otologic surgeon reviewed the three-dimensional reconstructions of the auto-segmented anatomy from all 25 testing datasets and found all yielded expected anatomic geometry. Seven experts assessed the manual and computer-generated segmentations superimposed on 4 TBCT blinded to the method of segmentation used. Each expert scored the segmentations according to the accuracy of the labeled structures. The mean reviewer scores for all the segmentations were similar for the manual and auto-segmentation methods (4.2 vs 4.3, p = 0.91), and no statistical significance was found in the analysis of each structure separately (Table 4).

**Table 4:** Expert reviewer ratings for the manually segmented and autosegmented temporal bone computed tomography scans. SD: Standard deviation.

Method	Manual		Auto		T-test
Structure	Mean accuracy score (1–5)	SD	Mean accuracy score (1–5)	SD	
Otic capsule	4.3	0.9	4.3	0.5	1
Inner ear	3.8	0.9	4.1	0.9	0.37
Ossicles	3.8	1	4	1	0.55
Facial nerve	4.6	0.5	4.4	0.5	0.44
Sigmoid sinus	4.8	0.5	4.5	0.7	0.17
Average	4.2		4.3		0.91

## Discussion

Otologic surgery is challenging given its small surgical field and complex interrelationships of bone and vital neurovascular structures. Clinicians face these challenges regularly when treating diverse conditions such as hearing loss, infections, and tumors of the temporal bone. The required knowledge of radiologic and surgical anatomy, as well as intraoperative 3D anatomical awareness, is achieved through extensive training, which takes considerable time and hands-on experience.

Computed tomography plays a central role in both the diagnosis and treatment planning of ear conditions. Although the datasets are volumetric, they are traditionally presented as a series of 2D multiplanar images to the surgeon, demanding experienced mental processing to transform these images to the required 3D representation. Virtual reality simulation systems can take advantage of reconstructed 3D models of anatomical structures and present them similarly to what is expected during surgery. It is reasonable to hypothesize that resulting virtual surgical rehearsal can result in a surgeon’s greater anatomic understanding, and ultimately improved patient safety. Intraoperative augmented reality may also help surgeons manage anatomic complexity^4,23^. The segmentation of key structures for these applications has been a limiting step due to the time and effort needed for this labor-intensive task.

Machine learning, particularly DL, which uses convolution neural networks, is promising in the automatic labeling of anatomical structures from clinical imaging because it is able to extract patterns that are not always readily apparent to the human eye. The growth of DL automatic segmentation has advanced with the availability of software toolkits such as the Clara SDK toolkit and the AIAA extension for 3D Slicer software^19,20^. The former facilitates the implementation of the computational environment for the training of algorithms and construction of automatic prediction models, while the latter provides a user-friendly interface to conduct segmentation predictions on robust research-oriented software.

The development of specific DL algorithms for three-dimensional data provides similar results faster than 2D algorithms when applied to volumetric tasks^18,24,25^. With the commercial availability of high-performance GPUs, we are now able to perform segmentation on images with input resolutions as high as 256 × 256 × 240, sufficient for the accurate identification of otologic structures as small as the semicircular canals and ossicles. In our work, the adapted AH-Net model showed faster inference time and similar accuracy results compared to other well-established 3-D segmentation models (U-Net^26^ and ResNet^27^) (Fig. 3). This highlights the improved efficiency of such algorithms, which makes more feasible to scale the pipeline to handle high volumes. We noticed a slightly inferior objective performance of AH-Net in the predictions of the inner ear and ossicles compared to those from other architectures. This may reflect a trade-off between speed and performance, since the AH-Net yielded predictions in about half the time as the other methods in many cases. Despite this, the AH-Net produced acceptable segmentation results and were subjectively indistinguishable from those using other approaches. Future studies may elucidate specific features of CNN architecture to optimize the segmentation of given anatomical structures, considering both their geometry and adjacent relationships. At the completion of training, the mean DSC from cross-validation showed strong correlation with the manually segmented ground truth for the inner ear (0.86) with similarly good correlation for other structures.

**Figure 4:**
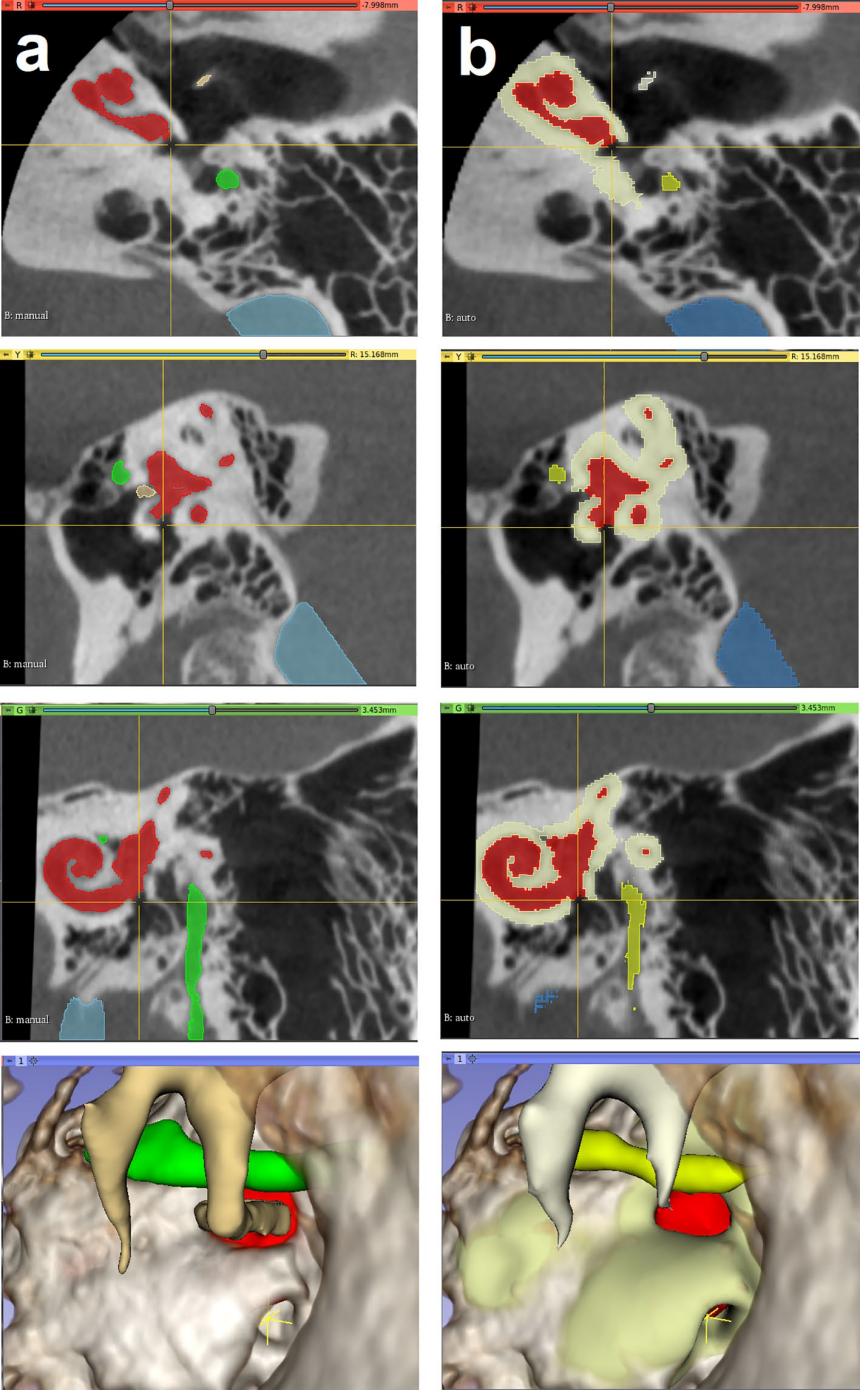
Round window niche (Crosshair) from a manual labeled scan (left—**a**) side by side with the auto-segmented CT dataset (right—**b**). From top to bottom, windows from 3D Slicer showing the axial, sagittal, coronal and 3D rendered view of the middle ear. Inner ear (red), ossicles (ivory); facial nerve (yellow), sigmoid sinus (blue) and otic capsule (green).

**Figure 5:**
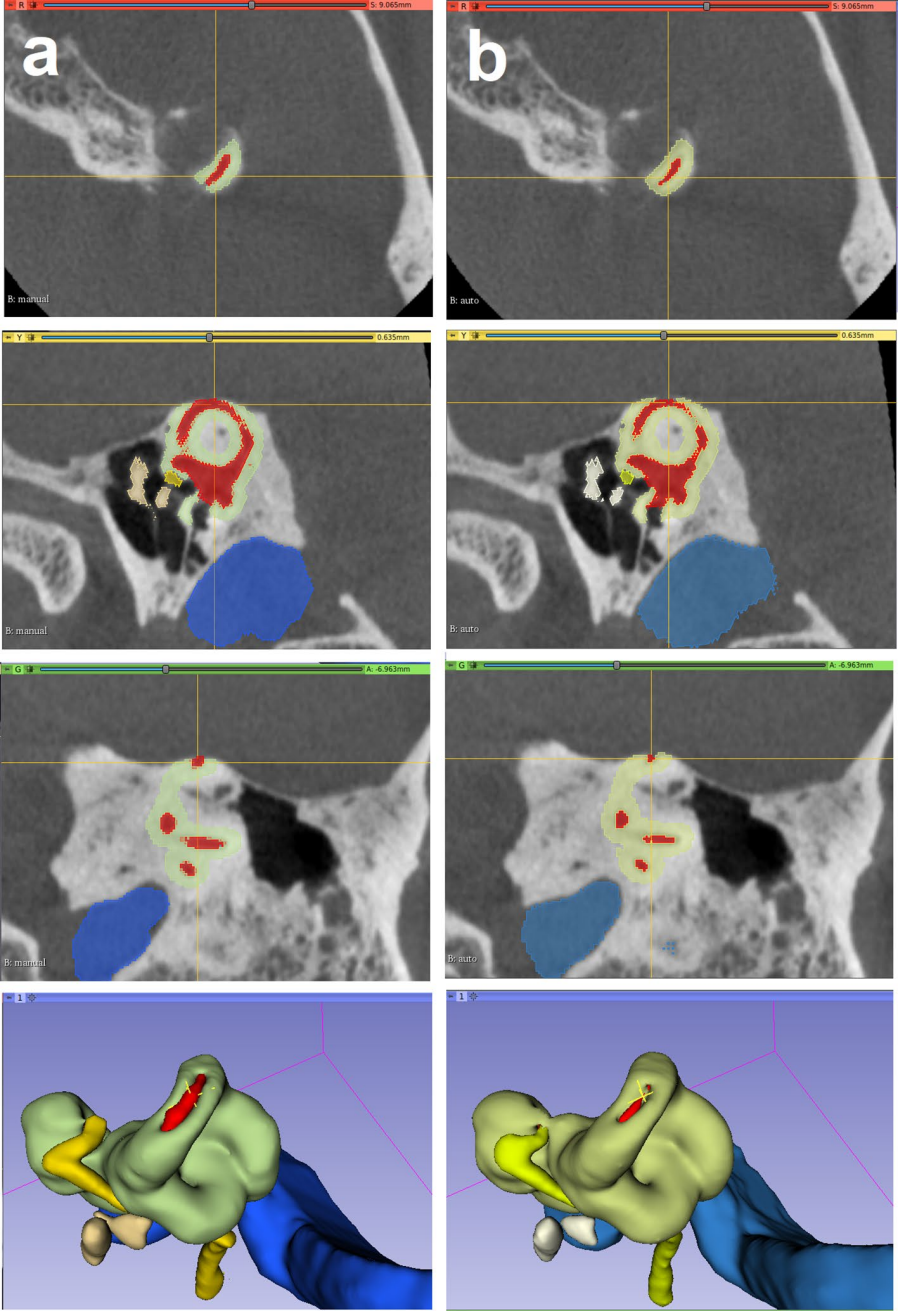
Superior semicircular canal dehiscence as demonstrated from manual (**a**) and auto-segmented (**b**) CT dataset seen in 3D Slicer. Note the lack of bone covering the balance canal (at the crosshair). Understanding the location and size of such a defect can facilitate surgical planning. Inner ear (red), ossicles (ivory); facial nerve (yellow), sigmoid sinus (blue) and otic capsule (green).

The otic capsule is the hard bone that surrounds the inner ear. It is more radiodense than surrounding bone, although not easily identified with the window levels usually used during clinical CT review. Our method of segmenting the otic capsule leverages this difference in radiodensity and identifies all voxels adjacent to the autosegmented inner ear with a HU values indicative of compact bone. This approach yielded satisfactory visual results (Fig. 4). This modeling produced valuable spatial information of the otic capsule. In particular, the resulting identification of the round window niche could potentially help surgeons predict and plan approaches to the inner ear during cochlear implantation (Fig. 4). Another advantage of the method is the ability to identify and evaluate pathologic otic capsule defects, such as those occurring in superior semicircular canal dehiscence (Fig. 5). Knowing the size and location of such deficits in relation to surrounding anatomy may help select optimal surgical approaches (e.g., transmastoid vs middle fossa). We intend to investigate the clinical utility of this method in future studies.

The segmentations from the training and testing set showed overall similar results for the DSC, suggesting that the prediction model was not overfitting the data. We implemented a prediction pipeline in 3D Slicer platform, which infers the structure segmentation and improves it by maximizing its contours as well as by removing extraneous voxels (false-positives). When applied to the testing set, the DSC for the post-processed segmentations were slightly increased compared to the coefficients of the raw prediction of the same dataset. This likely indicates a benefit from such post-DL refinement, and it is likely that additional post-processing techniques can be developed to improve the accuracy of DL auto-segmentation.

Our findings in the objective analysis are superior to those of other recently published automated segmentation methods for the inner ear, ossicles^28^ and facial nerve^5,15^. Despite these encouraging metrics, there is still variability between the structures. It is likely that auto-segmentation of the inner ear is facilitated by the fact that it is a fluid filled structure almost entirely surrounded by the radiodense hard bone of the otic capsule, providing consistent contrast with its surroundings. This is unlike the facial nerve, which has multiple interfaces with soft tissue, air, and heterogeneous bone throughout its convoluted course. This, coupled with its long path and small volume, likely reduces auto-segmentation accuracy. Similarly, the small size of the ossicles and absence of clear contours for the sigmoid sinus in the non-contrast TBCT used may have limited their DSC values.

Structure volumes were similar between the manual and autosegmented datasets with an average of 101% for all structures. For the facial nerve, we found a higher variance (SD = 24.3%) with the automated segmentation, which is likely influenced by its complex shape and the variability present in the manual segmentations.

While the DSC and volumetric comparison provide indices of similarity, the AHD represents the metric error between the different segmentation methods^29^. Therefore, a smaller AHD demonstrates a smaller error between the auto-segmentation and our ground truth manual segmentations. The AHD values indicated minimal errors for the inner ear, ossicles and facial nerve (0.25, 0.21 and 0.24 mm respectively). For the sigmoid sinus, the AHD was 0.45 mm. Although higher than the AHD of the other structures, it is still within an acceptable range for a structure with such a large volume.

Manual segmentation required focused user attention for an average of 211 s for each structure, whereas the automated method took only 2.7 s per structure. This 90-fold reduction in segmentation time demonstrates the significant efficiency gains of auto-segmentation and highlights the potential scalability for clinical use of the proposed pipeline. This will facilitate the integration of patient-specific anatomic models into planning and simulation systems by both surgeons and researchers^30^.

In the blinded expert review, both manual and automated segmentations were rated highly for accuracy. The ratings of manual and automated segmentations were similar for all structures, with no statistical significance apparent between the methods. These results further support the potential application of our method for clinical and research tasks previously requiring lengthy manual segmentation.

One limitation of our study is the relatively small size of the training dataset used to build the DL model. This was primarily the result of the laborious methods of manual segmentation. Due to the random assignment of the imaging studies to training and testing sets, it is possible that different ears from the same subject were included in both the training and testing set. Such inclusion could reduce the independence of these sets. However, in most cases there is considerable anatomic variability between different ears of a given subject^31–33^. We therefore expect that this possibility will do little to reduce the robustness of the model.

We anticipate that our current approach will provide the basis for obtaining many more segmented datasets, which can then be reviewed, refined and reintroduced into the training dataset. Through this process, our approach should yield even greater accuracy over time. Another limitation is that all of our training CT data were derived from a single institution and most contained normal anatomy. We hope that in the future, we can incorporate studies from a larger variety of scanners and include additional pathology to improve the robustness of the model. It remains to be seen how pathologic variation will affect the auto-segmentation process. The inclusion of multi-modality imaging (such as contrast enhancement and/or MRI data) may also be used in the future to improve the auto-segmentation process.

Our end-to-end approach to temporal bone segmentation provides a number of potential advantages over prior attempts since it: (1) is entirely automated and requires no user input that could introduce variability, (2) yields segmentation results similar to those created by trained experts, (3) is considerably faster than manual alternatives, making it more feasibly integrated into clinical workflow, (4) is built upon state-of-the-art machine learning techniques available through an open-source, freely available platform, ensuring the potential future refinement, and (5) is built from standard clinical scans, allowing for continual optimization with the addition of more varied patient datasets. We hope that our auto-segmentation platform will accelerate the segmentation and utility of temporal bone imaging studies and advance efforts towards multi-institutional collaboration in the construction of more robust and accurate models.

## Conclusion

We demonstrate a promising automated pipeline for segmentation of key otologic structures from clinical temporal bone CT datasets with good accuracy, as measured by the mean Dice score, average Hausdorff distance, volumetric comparison, and expert validation.

There remain areas in which the accuracy of this approach needs to improve prior to clinical use. However, these early results are quite encouraging, and with additional optimization, the results should greatly assist in the generation of anatomic datasets for clinical AR and VR applications, as well as other technologies that can improve surgical planning, workflow, and outcomes. Our future studies will focus on addressing some of these efforts.

## Methods

The study was approved by Stanford University Institutional Review Board (No. 38946), which granted a waiver of informed consent, since this retrospective study was conducted with anonymized data. All methods were carried out in accordance with institutional guidelines and regulations. TBCT clinical datasets with either 0.125 mm or 0.25 mm resolution were collected from 150 imaged ears (81 patients). A crosswalk to the true identifier of each TBCT avoided duplicates on the dataset. Patients with normal anatomy as well as those displaying alteration by pathology (e.g., chronic mastoiditis, postoperative changes, etc.) were included. Images with motion artifact or metal artifact that limited the evaluation were excluded. Manual segmentation of key structures of the 150 TBCT (Fig. 1) was conducted by a trained otologic surgeon and reviewed by an experienced neurotologist. 3D Slicer (www.slicer.org), an open-source medical image analysis software^34^ was used to create label maps of the inner ear, facial nerve, ossicles, and the sigmoid sinus from these CT datasets.

125 randomly selected volumes were set for the training set. The 25 remnant scans were included in the testing set for the prediction pipeline. Pre-transforms on the training TBCT included resizing and resampling images and labels previously at 512 × 512 × 480, 0.125 mm to 256 × 256 × 240, 1 mm voxel resolution, converting all datasets as right sided for standardization. The images had their intensity rescaled to a range between 0.0 to 1.0 by cropping the original image intensity between − 500 and 2000 HU. In order to increase the variability of our training sample and overcome the great variability in scans protocols for posterior inferences, data augmentation was achieved during the training by oscillating the intensity of the images in 10% of magnitude (intensity multiplied by a random factor between 0.9 and 1.1 in 10% of probability on every epoch) as well as horizontal flipping the images and labels in 50% of probability. Three 3D CNN (U-Net, ResNet and AH-Net) with Adam optimization algorithm^35^ were adapted for the task and trained on a Linux-based computer (Ubuntu 18.04) with AI capabilities in an environment facilitated by Clara SDK, an open-source toolkit to leverage deep learning projects in healthcare^19^.

U-Net^36,37^ has since been described for use in segmentation of biomedical images. Its architecture resembles the letter U, as it is characterized by the symmetrical sequence of contraction blocks followed by expansion blocks. The contraction comprises of convolution layers that reduce the size of the image and capture relevant features for its identification, whereas the expansion path includes a sequence of up-convolutions and concatenations that group feature with map information to allow image segmentation with accurate location. Since its advent, ResNet^22^ has been one of the most popular architectures in image segmentation applications. Its implementation of residual learning blocks as shortcut connections between the convolution layers targets to avoid the degradation of accuracy as the depth of the network increases. AH-Net^25^ is implemented in the ResNet structure, therefore it also has its contracting and expansion paths called encoder and decoder respectively. Radiological images in the clinical context are often acquired and presented as anisotropic volumes, and important information can be contained between sequential cuts. To deal with this limitation and improve the between-slice information prediction, AH-Net uses a hybrid encoder derived from a 2D network and transformed into a 3D encoder as described in Liu et al.^25^. The AH-Net architecture also features dynamic input shape which aims to increase the inference speed.

Using 24 GB of GPU RAM (two Titan XP GPU—NVIDIA, Santa Clara-CA), the dataset of manually-segmented CT images was used to train the DL algorithms for 2000 epochs with the initial learning rate set to 10^–4^. Along the training process the learning rate was set to a step decay by a third every 400 epochs and fivefold cross validation of the training set was performed to prevent overfitting.

The final prediction models were integrated into the 3D Slicer Nvidia AIAA client extension^20^ to perform the auto-segmentation of the inner ear, facial nerve, sigmoid sinus, and ossicles on the testing set (n = 25) (Fig. 2). We also created an automated model of the otic capsule by growing the margins of the auto-segmented inner ear within a compact bone HU threshold range (650–2500 HU).

The prediction pipeline was implemented in a 3D Slicer module to increase the inference speed, remove eventual noise, and maximize the contours of the segmentations. This step of post-processing included the resample of the volume to an isotropic spacing of 0.25 mm prior to the inference and automated margin correction of the predicted segmentations. Within the HU threshold range for the reference of each structure (e.g., bone HU threshold ranged from 100 to 2500 for ossicles, and soft tissue threshold ranged from -400 to 550 for the inner ear and facial nerve), the segmentation margins were maximized by its growing or shrinking within a certain Hounsfield unit range for each structure and small islands of discontinuous and incorrectly predicted voxels were excluded. The code for this process is publicly available^38^.

The objective assessment of the prediction model was done in the testing set using the mean Dice similarity coefficient (DSC), the average Hausdorff distance (AHD)^29^ and volumetric comparison. The time for manual segmentation and auto-segmentation was measured for each structure on the testing set, and it was used as metric to analyze the efficiency of the segmentation process.

For clinical validation, seven experts evaluated the segmentations of each structure on each of four TBCT (two expert manually segmented datasets and two automatically segmented datasets). Two head and neck radiologists and five neurotologists blinded to the method utilized (manual or automated) answered a 5-point Likert scale questionnaire to assess the accuracy of the segmentations (Supplementary Table S1). Two-tailed Student’s t-test was used to evaluate the similarities of the mean scores for each segmented structure. Estimates were considered statistically significant at α = 0.05.

## Supplementary Information

Supplementary Information 1.

Supplementary Information 2.
