# Supplementary material for: Fully automated preoperative segmentation of temporal bone structures from clinical CT scans
Source: Sci Rep. 2021 Jan 8;11:116. doi: 10.1038/s41598-020-80619-0 (PMC7794235; doi:10.1038/s41598-020-80619-0)
Supplement: Supplementary file 1 — Supplementary Information 1. [file 41598_2020_80619_MOESM1_ESM.pdf]

Title: Fully automated preoperative segmentation of temporal bone structures from clinical CT scans

Authors:

Neves CA[1], Tran ED [2], Kessler IM[1], Blevins NH [2]

[1]University of Brasilia, Faculty of Medicine, Brasília - DF, Brazil

[2] Stanford University School of Medicine, Otolaryngology Head & Neck Surgery, Stanford-CA, United States

Supplementary table S1

Questionnaire in Likert scale completed by experts when assessing the accuracy of the segmentation.

QUESTIONNAIRE

Please answer each item using the Likert scale according to your evaluation of the Temporal bone CT datasets.

Accuracy of the segmentation of the middle and inner ear structures from CT scans

Dataset #	Bad				Good
Otic capsule	1	2	3	4	5
Inner ear	1	2	3	4	5
Ossicles	1	2	3	4	5
Facial Nerve	1	2	3	4	5
Sigmoid sinus	1	2	3	4	5
