# Supplementary material for: Fully automated preoperative segmentation of temporal bone structures from clinical CT scans
Source: Sci Rep. 2021 Jan 8;11:116. doi: 10.1038/s41598-020-80619-0 (PMC7794235; doi:10.1038/s41598-020-80619-0)
Supplement: Supplementary file 2 — Supplementary Information 2. [file 41598_2020_80619_MOESM2_ESM.pdf]

Title: Fully automated preoperative segmentation of temporal bone structures from clinical CT scans

Authors:

Neves CA[1], Tran ED [2], Kessler IM[1], Blevins NH [2]

[1]University of Brasilia, Faculty of Medicine, Brasília - DF, Brazil

[2] Stanford University School of Medicine, Otolaryngology Head & Neck Surgery, Stanford-CA, United States

SUPPLEMENTARY TABLE S2

More details of the training parameters from the High level API interface in Clara SDK.

AH-Net details

- Optimizer: Adam
- Total epochs: 2000,
- Number of epochs for validation: 10,
- Learning rate: 0.0001,
- Multi-GPU: true,
- Learning rate policy : Step decay:
  - Decay ratio: 0.33,
  - Decay frequency: 400 epochs,
- Segmentation architecture: AH-Net
  - Number of spacial dimensions: 3
  - Dropout: false
  - Final activation layer: Softmax
- Dynamic_input_shape : true,
- Scale Intensity Range:
  - Fields: "image",
  - "a_min": -500,
  - "a_max": 2000,
  - "b_min": 0.0,
  - "b_max": 1.0,
  - "clip": true
- Scale Intensity Oscillation:
  - Fields: "image",
  - "magnitude": 0.10
  - “probability”: 0.10

ResNet

- Optimizer: Adam
- Total epochs: 2000,
- Number of epochs for validation: 10,
- Learning rate: 0.0001,
- Multi-GPU: true,
- Learning rate policy : Step decay:
  - Decay ratio: 0.33,
  - Decay frequency: 400 epochs,
- Segmentation architecture: 3D ResNet
  - Encoder layer number and ResNet block number of every layer: 1,2,2,4
  - Decoder layer number and ResNet block number of every layer: 1,1,1
  - Convolution kernel number of first layer: 16,
  - Dropout rate for several layers in training: 0.0,
  - Final activation layer: Softmax
- Dynamic_input_shape : false,
- Scale Intensity Range:
  - Fields: "image",
  - "a_min": -500,
  - "a_max": 2000,
  - "b_min": 0.0,
  - "b_max": 1.0,
  - "clip": true
- Scale Intensity Oscillation:
  - Fields: "image",
  - "magnitude": 0.10
  - “probability”: 0.10

U-Net

Optimizer: Adam

Total epochs: 2000,

Number of epochs for validation: 10,

Learning rate: 0.0001,

Multi-GPU: true,

Learning rate policy : Step decay:

Decay ratio: 0.33,

Decay frequency: 400 epochs,

Segmentation architecture: U-Net

Number of filters in each encoder layer: "32,64,64,64",

Number of filters in each decoder layer: "64,64,64,64,64,32,32",

Dropout rate for several layers in training: 0.0,

Final activation layer: Softmax

Dynamic_input_shape : false,

Scale Intensity Range:

Fields: "image",

"a_min": -500,

"a_max": 2000,

"b_min": 0.0,

"b_max": 1.0,

"clip": true

Scale Intensity Oscillation:

Fields: "image",

"magnitude": 0.10

“probability”: 0.10
